# The DNA of Bacteria of the World Ocean and the Earth in Cosmic Dust at the International Space Station

**DOI:** 10.1155/2018/7360147

**Published:** 2018-04-18

**Authors:** T. V. Grebennikova, A. V. Syroeshkin, E. V. Shubralova, O. V. Eliseeva, L. V. Kostina, N. Y. Kulikova, O. E. Latyshev, M. A. Morozova, A. G. Yuzhakov, I. A. Zlatskiy, M. A. Chichaeva, O. S. Tsygankov

**Affiliations:** ^1^Federal Research Center of Epidemiology and Microbiology named after Gamalei, Moscow, Russia; ^2^Peoples Friendship University of Russia (RUDN University), 6 Miklukho-Maklaya St, Moscow 117198, Russia; ^3^Central Research Institute of Machine Building, Korolev, Russia; ^4^Dumanskii Institute of Colloid and Water Chemistry, National Academy of Sciences of Ukraine, Kiev, Ukraine; ^5^Korolev Rocket and Space Corporation «Energia», Korolev, Russia

## Abstract

Cosmic dust samples from the surface of the illuminator of the International Space Station (ISS) were collected by a crew member during his spacewalk. The sampler with tampon in a vacuum container was delivered to the Earth. Washouts from the tampon's material and the tampon itself were analyzed for the presence of bacterial DNA by the method of nested PCR with primers specific to DNA of the genus* Mycobacteria*, DNA of the strains of capsular bacteria* Bacillus*, and DNA encoding 16S ribosomal RNA. The results of amplification followed by sequencing and phylogenetic analysis indicated the presence of the bacteria of the genus* Mycobacteria* and the extreme bacterium of the genus* Delftia* in the samples of cosmic dust. It was shown that the DNA sequence of one of the bacteria of the genus* Mycobacteria* was genetically similar to that previously observed in superficial micro layer at the Barents and Kara seas' coastal zones. The presence of the wild land and marine bacteria DNA on the ISS suggests their possible transfer from the stratosphere into the ionosphere with the ascending branch of the global electric circuit. Alternatively, the wild land and marine bacteria as well as the ISS bacteria may all have an ultimate space origin.

## 1. Introduction

The attention of researchers has always been attracted by the question if there are any microorganism's markers in the conditions of outer space. At the beginning of the 20th century Svante Arrhenius formulated the hypothesis of panspermia [1, 2], in which he suggested an extraterrestrial source of life. The development of this hypothesis gave rise to an idea of a possible way of transportation of bacteria and spores through interplanetary and interstellar space. Despite the unpopularity of Arrhenius hypothesis, it had and still has significant influence on the development of entire areas in the fields of biological and space research. Hoyle and Wickramasinghe [3–5] have reexamined the Arrhenius hypothesis using new data. A number of researchers have made an effort to investigate panspermia [6, 7] and have paid much attention to the exploration of survival mechanisms of living organisms in the interplanetary space [8–10]. Survival of different types of microorganisms to a prolonged exposure in space was confirmed in experiments, which were performed on the American and Japanese segments of ISS. Thus Antarctic rocks colonized by lichens and fungi (*Stihosotskus* sp. and* Acarospora *sp.) were placed for 1.5 years on the ISS, simulating conditions on Mars, and then investigated by molecular methods [11]. Scientists tested bacterial endospores in the space experiment “molecular strategies of microbial adaptation to different space's and planetary climate conditions.” It was determined that extraterrestrial ultraviolet (UV) emission of sun (*λ* ≥ 110 nm) and also Martian UV (*λ* ≥ 200 nm) are the most harmful factors. However, even after such treatment, there remained a small percentage of live spores [12]. Aerobiological studies have also shown the existence of active and dormant forms of bacterial and fungal spores in the stratosphere, for example,* Micrococcus luteum, Mycobacterium albus, Circinella muscae, Aspergillus niger*,* Papulaspora anomala, Penicillium notatum *[13, 14];* Bacillus simplex, Staphylococcus pasteuri, Engyodontium album *[15];* Deinococcus aetherius *[16]; and* Bacillus isronensis *[17]. Some researchers have also carried out experiments studying the stability of plant seeds in vacuum and in conditions close to those on Mars [18].

From the time of the first orbiting satellites [9, 19] to modern projects (20–24) experiments in open space have shown extraordinary, possibly months-long resilience and survival of different taxonomic groups in the damaging radiation of near space. Results of these experiments confirmed the possibility of organisms preserving viability in the open space for a minimum of two years, and this gave rise to an interest in the question of living organisms being transferred from the stratosphere to the near-Earth space [20, 21].

We might conjecture that viable forms or at least intact DNA can be transferred to the ISS orbit with the ascending branch of the global electric circuit (GEC) [22], which is discussed in the works of Williams and Rycroft [23, 24]. A possible mechanism for the transfer of dispersed particles from the stratosphere to the ionosphere has been recently proposed [25, 26], but this idea has not been worked out in detail yet. In this paper samples of space dust from the exterior surface of the ISS were subjected to PCR analysis followed by sequencing of the PCR products. The results obtained indicate the possibility of a mass transfer of the sea bacterial plankton (or its fragments) to the ISS orbit. Alternatively, the transfer of biological material from space to ISS and thence to the ground may be supposed.

## 2. Materials and Methods

### 2.1. Space Dust Sampling

Cosmonaut Misurkin A. A. (a crew member of the expedition 36) during his spacewalk (22.08.2013) collected the samples of space dust using the tampon from the sampler “TEST” (Central Research Institute of Machine Building). He took 2 probes of dust from the surface of the porthole VL2 of the small research module “Poisk” (MIM2): from the porthole's frame (sample number 1) and from the glass and from the border between glass and frame (sample number 2). The porthole VL2 MIM2 is faced forward, along the vector of the ISS velocity.

Prior to being sent to the ISS, the sampler “TEST” was sterilized in autoclave and with *γ*- radiation. After the dust was collected from the surface of the ISS, the tampon holder was screwed into the body of the sampler “TEST” in the open space, so that the swab remained in vacuum until sampler opening in the terrestrial laboratory. In an Earth-based laboratory, the tampon was taken out from the sampler in a laminar flow hood with 2nd degree of biological protection (complete isolation of the working area air).

### 2.2. Sample Processing in the Laboratory

Sample processing was conducted in a laminar flow hood with 2nd degree of biological protection. To avoid contamination, the manipulations were carried out with gloves and the operator was provided with a mask and disposable clothing. We made a suspension from tampons in sterile distilled water (50 ml). We also obtained washes with sterile distilled water (50 ml) from the inner surface of sampler's slots for tampons. Then the samples were concentrated using special tubes Amicon Ultra-15 (Millipore), and 400 *μ*L of every sample was used for total separation of nucleic acids. The accumulating membrane was also crushed and analyzed.

### 2.3. Nucleic Acids Separation

Probes themselves and also the samples of collecting membrane were suspended in 6 M solution of guanidine thiocyanate and isolated using the inorganic carrier SiO_2_. Elution was carried out into 0.03 cm^3^ of deionized water.

### 2.4. Polymerase Chain Reaction (PCR)

PCR to determine the bacterial plankton and the gene coding for 16S ribosomal RNA was performed in a volume of 0.025 cm^3^. PCR reaction mixture (0.02 cm^3^), containing 67 mM Tris-HCl рН 8.8 (at 25°C), 16.6 mM (NH_4_)_2_SO_4_, 0.01% tween-20, 1.5 mM MgCl_2_, 0.2 mM of each dNTPs, 10 pmol of each primer, and 1.25 units of Taq DNA polymerase, was added to 0.005 cm^3^ of DNA. We used universal primers F27/R1493 for the amplification of the 16S ribosomal RNA. We also used two primer pairs MTG1/MTG4 external and MTG1/MTG4 internal to determine the bacterial plankton gene. Specific bacteria encystation gene primers were utilized to detect the* Bacillus anthracis *DNA. In all cases we used nested PCR with the technique of “Hot Start” and antibodies to Taq DNA polymerase. Temperature conditions of amplification were as follows: 95°C-30 seconds (for the first and last cycle 1 min); 62°C-30 seconds; 72°C-30 seconds (for the first and last cycle 1 min). The ratio of amplification-reamplification cycles in nested PCR was 25 : 36.

### 2.5. PCR Fragment Sequencing

The sequencing was performed with the primers which were used for PCR. We conducted sequencing of fragments on two chains. The PCR products were purified in special gel purification kit (Geneclean) and then sequenced. Determination of the primary sequence was carried out on an automated DNA sequencer (ABI 377, USA). Primary sequence was analyzed using computer programs MacVector/AssemblyLign (Oxford Molecular Group, USA).

### 2.6. Analysis of the Nucleotide Sequences

The sequences were analyzed using the following programs: BLAST 2.7.0+ (https://blast.ncbi.nlm.nih.gov/Blast.cgi), DNAStar's MegAlign, and methods MacVector/AssemblyLign (Oxford Molecular Group, USA). Phylogenetic dendrogram was constructed using ClustalV and ClustalW methods.

## 3. Results

**Figure 1:**
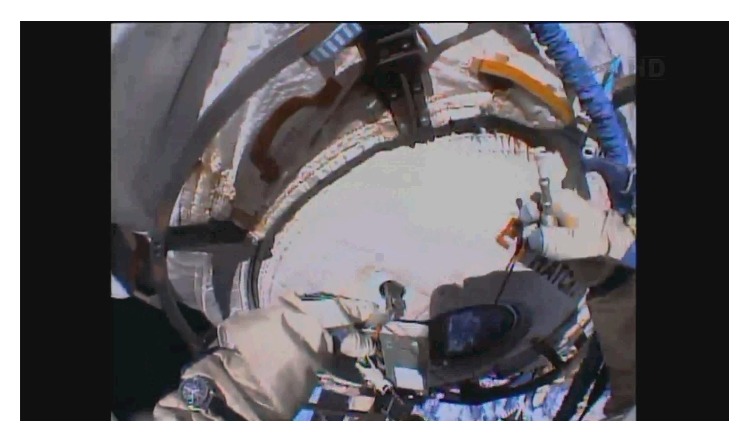
The process of sampling with sampler “TEST.” Astronaut holds the body of sampler “TEST” in his left hand and the tampon holder screwed out in the open space in the right hand.

Previously, researches have already collected cosmic dust on the ISS, trying to find the ionosphere bacteria from the lower atmosphere. For this task, in the experiment *«*Tanpopo*»* a special collector of cosmic dust on the basis of hydrophobic aerogel was constructed on the Japanese segment of the ISS [27]. The absence of PCR product in the samples of *«*Tanpopo*»* mission can be explained by the short duration of exposure and the location of the sampler. We guessed that a surface of the ISS is a more effective trap for the space dust, because it gathers the dispersed particles from the vacuum, just in the same way as the dust particles, including bacterial and fungal spores, are adsorbed from the gas phase on the surface of aircraft [28, 29]. Therefore, the most suitable for this purpose was the oncoming flow MIM2, which was docked to the zenith port of the ISS module “Zvezda” eight years ago (10.11.2009). This decision was prompted by the experimental data from Central Research Institute of Machine Building that showed organic contamination on the surface of ISS, which certainly facilitates the adsorption of cosmic dust particles.  Figure 1 shows the process of sampling from the exterior of ISS.

**Figure 2:**
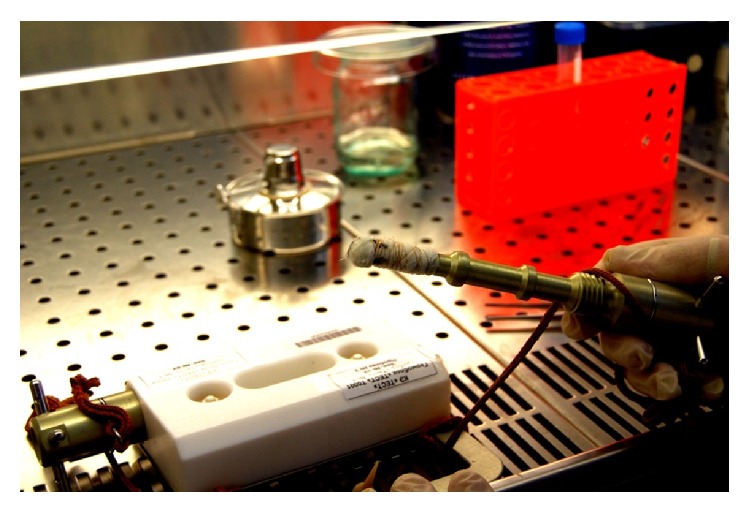
The process of the sampler “TEST” opening in the lab in a laminar flow hood with protection degree II. Room where the sample was processed had been pretreated with UV for 24 hours.

It must be emphasized that, to avoid drift of terrestrial bacteria into space, the sampler “TEST” was sterilized in an autoclave and with *γ*- radiation. After the dust was collected from the surface of the ISS, the astronaut screwed the tampon holder into the body of the sampler “TEST” in the open space, so that the swab remained in vacuum until the sampler was opened in the terrestrial laboratory, which was also important to avoid nucleic acids contamination.  Figure 2 shows the process of the sampler opening in the laboratory of molecular diagnostics.

Then we described the dispersed composition of the samples by the method of dynamic light scattering (DLS) [30].  Table 1 shows the characteristics of the disperse composition of swabs. It should be noted that we visually observed 2 black streaks on the tampon number 2.

**Figure 3:**
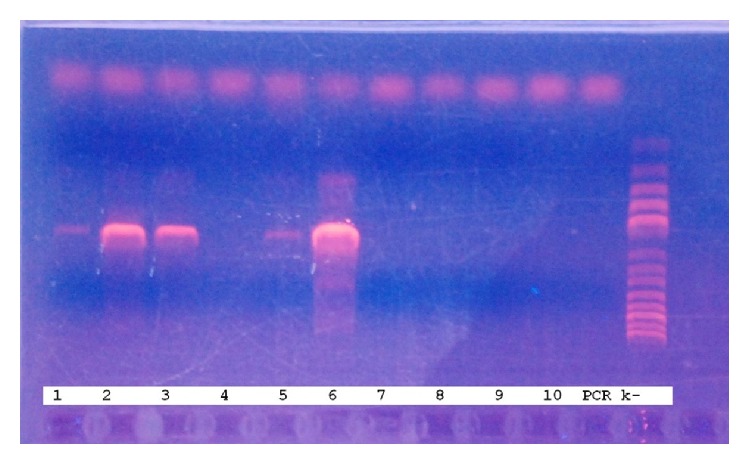
The results of amplification of nucleic acids extracted from the samples using universal primers specific for the 16S rRNA gene. Lane 1: tampon number 1, 2: washout from the cavity number 1, 3: membrane of tampon number 2, 4: washout from the tampon number 2, 5: membrane of washout number 2, 6: washout from the cavity number 2, 7: membrane of washout number 1, 8: tampon number 2, 9: tampon number 2, 10: membrane of tampon number 1, 11: negative control (PCR k−). Molecular weight markers (step 100 bp.) are applied on the last lane.

DNA encoding 16S ribosomal RNA gene was detected in both samples (washouts from the sampler cavity and the tampon) (Figure 3).

The amplified fragment of unknown DNA of 803 base pairs was sequenced. The nucleotide sequence of this PCR product fragment is shown at Table 2.

Phylogenetic analysis of the nucleotide sequence with the BLAST 2.7.0+ program and NCBI GenBank® DNA sequence databases showed a 100% identity with the genus* Delftia*, family Comamonadaceae, order Burkholderiales [31]. The genus taxonomic position is https://www.ncbi.nlm.nih.gov/Taxonomy/Browser/wwwtax.cgi?mode=Info&id=80865&lvl=3&lin=f&keep=1&srchmode=1&unlock.

Genus* Delftia* can be found in a variety of habitats (soils, bottom sediments, active slits, and natural waters), including marine ecosystems (http://www.marinespecies.org/aphia.php?p=taxdetails&id=571139).

Apart from the presence of* Delftia*'s DNA, we found in the samples of cosmic dust the DNA of the genus* Mycobacteria*, some species of which make up to 40% of the biomass of marine heterotrophic bacterial plankton.

Due to the importance of the results showing the presence of wild bacterial DNA in cosmic dust, we repeated the collection of cosmic dust on the ISS surface many times during several years (2013–2016). Summarized results of bacterial DNA detection in the samples of cosmic dust are shown in Box 1. During the experiments in open space during 2013–2016, more than ten probes of cosmic dust were collected. It is very important that the DNA of the same microorganisms (*Delftia *sp.*; Mycobacteria *sp.; Archaebacteria) was repeatedly found at different sites of the station in different years.

At the boundary “glass-frame” we discovered DNA of* Mycobacteria *sp. also attendant to bacterial plankton. We sequenced the DNA fragment, obtained after PCR with primers specific to gene of* Mycobacteria *sp. The nucleotide sequence of the PCR fragment is shown at Box 2.

**Figure 4:**
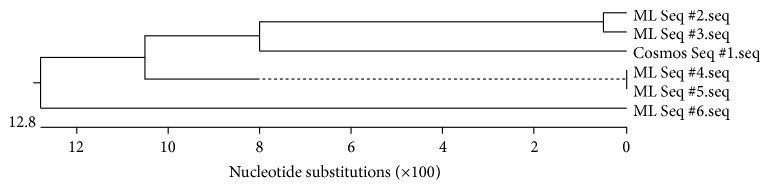
Phylogenetic dendrogram on the basis of the gene fragment obtained with universal primers specific to the* Mycobacteria *sp. DNA, Cosmos Seq # 1: the nucleotide sequence of a PCR product from the space dust. ML Seq # 2–ML Seq # 6: the nucleotide sequences of PCR products from the samples of sea surface microlayer.

The resulting nucleotide sequence was compared with the DNA sequences of* Mycobacteria *sp., obtained earlier from the samples from the coastal and marine surface micro layer in the Russian Western Arctic areas. The results of phylogenetic analysis are presented in Figure 4.

The data presented in Table 2 demonstrates that the DNA of terrestrial microorganisms on the surface of the ISS is not an accidental fact. The presence of the sought-for DNA in the dust samples points to an undefined process allowing the particles of biological material to be transported to the ionosphere or, alternatively, to the idea that common terrestrial bacteria are constantly being resupplied from space [4, 5].

## 4. Discussion

The discovery in the present study of the DNA of the marine and terrestrial bacteria on the surface of the ISS requires an explanation of the transport mechanisms allowing living matter to reach a height of more than 400 km. The composition of DNA of detected biomaterial excludes single accidental contamination of terrestrial origin. Furthermore, sampling and processing procedures that were used reduced the likelihood of contamination to a minimum. The absence of contamination is supported by clean negative controls, which are very important when using molecular techniques.

**Figure 5:**
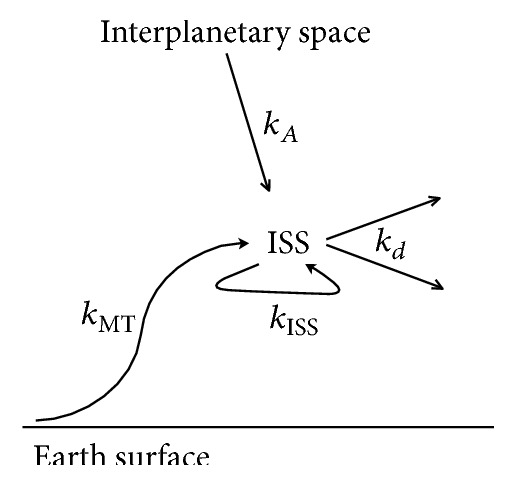
Possible ways of intact DNA (bacterial forms) transfer to the orbit of the ISS and from ISS surface. Kinetic constants of different transfer pathways of microorganisms are indicated as follows: *k*_*A*_: hypothetic Arrhenius' way from the interplanetary space, *k*_mt_: possible mass transfer of the dispersed matter from the Earth surface to the ISS orbit with the ascending branch of the global electric circuit, *k*_ISS_: possible contamination way from ISS internal volume to ISS surface, and *k*_*d*_: dissipation of the dust from the ISS surface to space.

Phylogenetic dendrogram (Figure 4) shows that the DNA sequence discovered in samples of cosmic dust has 93% identity with two of the five* Mycobacteria* sequences found in the samples from the marine surface micro layer and marine aerosol in the Russian Western Arctic. Of course, this phenomenon needs further exploration, perhaps sequencing of other genes of bacteria from this genus. However, we can state that the upward transport of bacterial DNA is one explanation of the close similarity. Aerosol transport, including living organisms, from the Earth's surface to the troposphere is well described [20, 21, 32–35]. The examples of bacterial genera, which can be attributed to the aerobes of troposphere, indicating the transport mechanisms from the surface of the ocean and land, are presented in the third line of Table 2. Transport from the troposphere to the stratosphere is also well described [36]. It should be stressed that the transfer of dispersed particles from the troposphere to the stratosphere is possible not only during/via catastrophic events (volcanic eruptions, forest fires), but also during the passage of vortex areas where the troposphere dramatically changes its height [34, 35]. The result of this transfer is a steady presence of a microbial community in the stratosphere (Table 2, line 2), thus raising the question of the upper boundary of the biosphere [13, 20, 21]. Transfer from the stratosphere to the ionosphere might be possible with the ascending branch of the global electrical circuit [23, 24] due to a variety of coupled mechanisms of turbulent electrothermodiffusion accompanied by mass transfer of dispersible material although these processes need further exploration [25, 26]. It is clear that there are different possible ways of dust transport to (and from) ISS not excluding the possibility of interstellar or interplanetary panspermia [3–5, 37] (Figure 5).

The presence of the DNA of wild terrestrial and marine species of bacteria on the surface of the ISS indicates their possible transfer from the stratosphere to the ionosphere with the ascending branch of the global electric circuit although the details of the process have not been worked out.

The bacteria survival during the transfer from the Earth's surface to the ionosphere is a particular problem. Intact DNA of the genera* Delftia *and* Mycobacteria *detected in this paper may indirectly indicate the intactness of the whole cell; otherwise the formation of thymine dimers would make the PCR conducting impossible. However, this issue needs further explorations. The question of a living organism's survival in open space has been carefully studied in space experiments [8–10], when the long-term stability of different taxonomic groups to corpuscular radiation, hard X-ray, and ultraviolet radiation was confirmed. In the experiment EXPOSURE-E maximum dose rates reached 0.4 mGy per day [38]. With this dose load and the median lethal dose for bacterial forms from hundreds of Gy [39, 40], the object transported from the Earth's surface into outer space has the potential to remain intact to radiation exposure for hundreds of years. It is worth noting that the intensity of generation of aqueous aerosol, including bacterial plankton [34, 35], from the Earth's surface can reach up to 6 Mt per year [41]. However the transport of such material above 30 km is not well documented. If there can be a migration or diffusion of bacterial particles above 30 km, this supports a hypothesis in which living matter is not* required* to be brought in with meteoritic or cometary material to the Earth [8, 37] but, on the contrary, is dissipated from the Earth biosphere into interplanetary space. The data also supports the possibility of microorganisms coming to the Earth from outside the Earth [3, 5].

## 5. Conclusion

We detected the DNA of bacteria of the genus* Mycobacteria *and extreme bacteria of the genus* Delftia* (family Comamonadaceae, order Burkholderiales) in the samples of cosmic dust. It was shown that the DNA sequence of one of the bacteria of the genus* Mycobacteria* was genetically similar to that previously observed in the sea surface microlayer at the Barents and Kara seas' coastal zones. The presence of DNA of wild land and marine bacterial genera on the ISS indicates their possible transfer from the stratosphere into the ionosphere with the ascending branch of the global electric circuit, alternatively to their entry into the Earth's environs from space.

## Figures and Tables

**Box 1:**
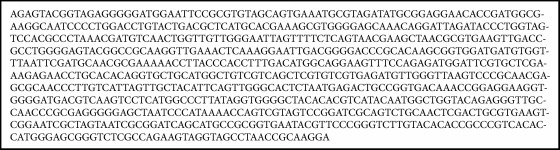
The sequence of the DNA fragment (size 803 bp.) obtained by amplification of samples from ISS surface with universal primers specific to the bacterial 16S rRNA gene.

**Box 2:**
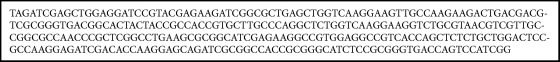
Nucleotide sequence of a PCR fragment obtained by amplification of space dust with universal primers specific to gene of *Mycobacteria *sp.

**Table 1:** Disperse composition of the washouts from the tampon and “TEST” sampler cavity.

Washout	The position of distribution maximum by the number of particles (*μ*m)/fraction (%)			
	1 fraction	2 fractions	3 fractions	4 fractions
Tampon no. 1	**0.04/0.1**	-	**0.69/98**	**5.3/1.9**
Cavity no. 1	0.17/22	0.33/75	-	4.8/3

Tampon no. 2	**0.11/32**	**0.44/36**	**0.84/20**	**5.5/12**
Cavity no. 2	0.10/38	0.26/59	-	5.0/3

**Table 2:** Presence of different bacterial DNA in the samples of cosmic dust from the surface of ISS.

Year	Location of dust sampling	Bacteria species/foreign substance
2013	Porthole frame of VL2 of the small research module 2	*Delftia *sp.
	Glass-frame border of the porthole frame of VL2 of the small research module 2	*Delftia *sp., DNA of *Mycobacteria *sp. (marine bacterioplankton)

2014	Cover of the porthole no. 13 of the service module	Organic matter, including peptides and flavonoids
	Porthole VL 2 of docking compartment 1	DNA of uncultivated soil bacteria from Madagascar and reservoirs
	The surface of photoelectron converters of solar panels	DNA of Archaebacteria

2015	Radiator surface of the thermoregulation system of the service module	DNA sequences close to the genomes of uncultured fungi
	Radiator surface of the thermoregulation system of the service module, between radiator tubes	DNA of Archaebacteria; DNA of fungi *Aureobasidium *sp.
	Brackets 2434 and 2435 of the service module	DNA sequence close to the plant genomes
	Porthole VL 1 of the docking compartment 1	DNA *of Erythrobasidium *sp.*, Cystobasidium *sp.*, Staphylococcus saprophyticus, Agrococcus jenensis*

2016	Radiator surface of the thermoregulation system (near porthole no. 8) of the service module	DNA of *Mycobacteria *sp. (marine bacterioplankton), fungi DNA of the genus *Bjerkandera*
